# Oligonucleotide Functionalised Microbeads: Indispensable Tools for High-Throughput Aptamer Selection

**DOI:** 10.3390/molecules201219766

**Published:** 2015-12-01

**Authors:** Lewis A. Fraser, Andrew B. Kinghorn, Marco S. L. Tang, Yee-Wai Cheung, Bryce Lim, Shaolin Liang, Roderick M. Dirkzwager, Julian A. Tanner

**Affiliations:** School of Biomedical Sciences, Li Ka Shing Faculty of Medicine, The University of Hong Kong, Pokfulam, Hong Kong, China; lewis-fraser@hku.hk (L.A.F.); andrew.b.kinghorn@gmail.com (A.B.K.); szelokt@hku.hk (M.S.L.T.); cheungw@hku.hk (Y.-W.C.); bl404@cam.ac.uk (B.L.); shaolin2@hku.hk (S.L.); rdirkz@hku.hk (R.M.D.)

**Keywords:** aptamer, SELEX, microbead, monoclonal bead, ePCR, FACS

## Abstract

The functionalisation of microbeads with oligonucleotides has become an indispensable technique for high-throughput aptamer selection in SELEX protocols. In addition to simplifying the separation of binding and non-binding aptamer candidates, microbeads have facilitated the integration of other technologies such as emulsion PCR (ePCR) and Fluorescence Activated Cell Sorting (FACS) to high-throughput selection techniques. Within these systems, monoclonal aptamer microbeads can be individually generated and assayed to assess aptamer candidate fitness thereby helping eliminate stochastic effects which are common to classical SELEX techniques. Such techniques have given rise to aptamers with 1000 times greater binding affinities when compared to traditional SELEX. Another emerging technique is Fluorescence Activated Droplet Sorting (FADS) whereby selection does not rely on binding capture allowing evolution of a greater diversity of aptamer properties such as fluorescence or enzymatic activity. Within this review we explore examples and applications of oligonucleotide functionalised microbeads in aptamer selection and reflect upon new opportunities arising for aptamer science.

## 1. Introduction

**Figure 1:**
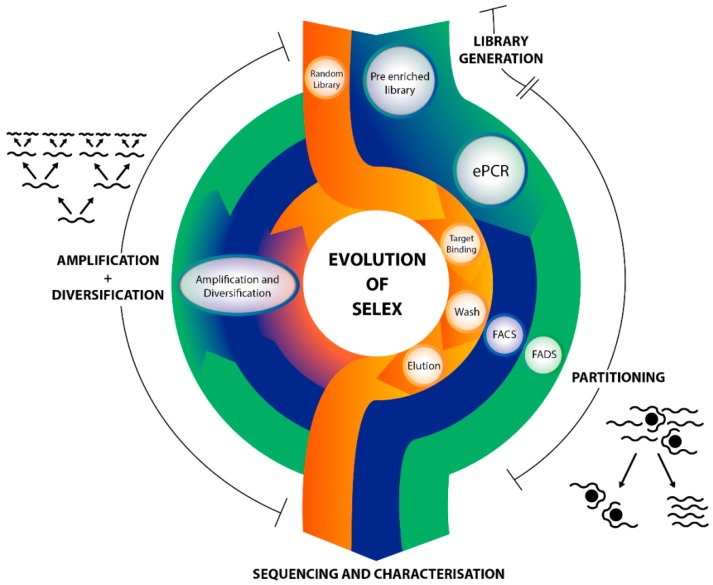
The evolution of SELEX. The SELEX cycle diagram compares the stages involved in a classical SELEX cycle to those which utilise monoclonal beads or compartmentalised droplets during selection. In classical SELEX (orange), PCR is used in library amplification. In the partitioning stage: target binding, wash, and elution steps are manually performed. In contrast monoclonal bead modified SELEX (blue) and droplet SELEX (green) both use ePCR for library amplification and high-throughput partitioning using FACS and FADS respectively.

Aptamers are synthetic oligonucleotides that fold into unique 3-D structures that specifically bind to their targets with high affinity. Since aptamers were first described by two independent research teams in 1990 [1,2], they have been rapidly adapted for various applications in research and biotechnology. Aptamers can be selected against a wide variety of targets including metal ions [3,4], small molecules [5,6], proteins [7,8], and even whole cells [9,10]. Aptamers offer multiple advantages when compared to the analogous protein based affinity reagents, antibodies. These advantages include greater stability, specificity, ease of chemical modification, lower production cost and less batch to batch variability [11]. Aptamers are typically selected from a random library through a process called systematic evolution of ligands by exponential enrichment (SELEX). Figure 1 (Orange) shows the basic method for obtaining an aptamer by SELEX. Generally, SELEX comprises the iterative steps of aptamer library binding, elution of bound species and pool amplification until aptamer candidates with desired criteria are selected. A random nucleic acid library is incubated with a target. Sequences which bind to the target are then partitioned and any non-binding or low affinity sequences are discarded. Binding sequences are eluted and amplified by the polymerase chain reaction (PCR) which will seed the next round of SELEX. Rounds are repeated until sequence enrichment occurs at which point aptamer candidates can be sequenced and characterised.

Classical SELEX is a well-established approach, however it is labour intensive and time-consuming. As is common of any evolutionary process, classical SELEX is sensitive to stochastic effects that influence the selection process. These effects can include loss of tight binding members, retention of weak binders or random fluctuations in amplification. In the first round of selection, there may be as low as one copy of any given aptamer candidate, resulting in the loss of ideal binders and unwanted selection of spurious sequences. Even though these effects were ignored in early SELEX numerical and theoretical simulation experiments [12,13], there is growing evidence to support that stochastic effects can substantially affect selection results, especially when dealing with complex targets [14,15]. As a result several techniques have been developed to improve selection. These involve the isolation and amplification of individual sequences such that sufficient binding events occur per sequence candidate to allow for detectable assays to be employed [16,17,18]. By assaying each individual member of the library, the stochastic effect is greatly reduced.

Microbeads are small amenable particles that are widely used in both industry and research. They comprise of solid, gel-like or vesicular particles with sizes ranging from nanometre to millimetre diameters [19]. Magnetic microbeads have been used extensively in classical SELEX experiments for immobilising selection targets, including our own experience [7,20,21]. This substantially simplifies the partitioning of target-bound and -unbound species during selection. Since the first application of magnetic microbeads for aptamer selection in 1996 [22], more sophisticated technologies which harness the potential of microbeads in optimising and streamlining SELEX experiments have been developed. Therefore this review will focus on the impact such microbead technologies have had on aptamer research.

Microbeads have the ability to be decorated with oligonucleotides of a single species. Despite inherent drawbacks associated with surface immobilisation of aptamers [23], monoclonal beads have greatly enhanced partitioning (Figure 1) during the aptamer selection process. In classical SELEX the partitioning process is made up of target binding, washing and elution (Figure 1, Orange), all of which are stochastic in nature and lead to loss of fit sequences. The notion of characterising every aptamer in an early round SELEX is daunting yet would eliminate the single molecule stochastic effect problems limiting the partitioning process. A promising method to limit these factors is monoclonal microbead technology. Flow cytometry or FACS technology is currently capable of sorting cells and beads at rates of up to around 10^8^ per hour [24]. By combining FACS technology with pre-enriched monoclonal bead libraries, each individual aptamer candidate can be assayed and partitioned automatically in a high-throughput manner (Figure 1, Blue) [18], thereby minimising the stochastic effects encountered in classical selection techniques. This transition from binding capture, vulnerable to stochastic effects, to FACS selection is a major step forward in terms of selection efficiency and efficacy. This improvement is best illustrated by the 1000 fold increase in binding strength achieved when aptamers for a certain target were selected using FACS instead of classical techniques [18]. This review therefore considers the various theoretical and experimental considerations involved in these new microbead-based selection technologies.

Aptamer research has primarily focused on discovery of oligonucleotide sequences with binding properties, however molecular binding represents a fraction of their functional potential. Examples from biology—including, most famously, the ribosome—exemplify the vast catalytic potential of oligonucleotides. Ribozymes, DNAzymes and XNAzymes have been developed *in vitro* to perform simple enzymatic functions whereby clever strategies need to be used to link product to reactant during the selection [25]. Microbeads show potential in developing higher complexity enzymatic interactions by combining monoclonal encapsulation and FADS technology (Figure 1, Green).

## 2. Functionalised Bead Generation

Methods for the generation of oligonucleotide-functionalised monoclonal microbeads have been pivotal in advancing aptamer selection techniques. These monoclonal beads can be produced using several different techniques, the most promising of which uses ePCR. To date, several examples of aptamer selections incorporating monoclonal beads generated by ePCR have been reported (Figure 1, Blue) [18,26,27]. This section therefore seeks to fully explore the different techniques involved in monoclonal bead generation for the application of aptamer selection and primarily focuses on the promising area of ePCR.

### 2.1. One-Bead-One-Oligonucleotide Library

Combinatorial chemistry has enabled the simple construction of monoclonal bead-based oligonucleotide libraries by the “split synthesis” method [28]. The “one-bead-one-compound” concept was originally developed for synthetic peptide library generation [29], and was subsequently expanded to the generation of oligonucleotide [30] and non-peptide oligomeric compounds [31]. These combinatorial libraries contain single beads each displaying 10^13^ copies of a single compound, thereby facilitating the assaying of single beads in downstream applications. The first one-bead-one-oligonucleotide library for SELEX was used to select synthetic phosphodiester-modified oligonucleotides against transcription factor NF-kB p50/p50 protein in combination with FACS screening [30,32]. However, application of combinatorial oligonucleotide library generation directly onto beads remains limited in SELEX since only a small library can be represented. Fully representing a 20-nucleotide sequence would require 10^12^ individual beads to be synthesised and assayed. Additionally just one round of selection can be performed on these chemically synthesised libraries which is a significant hindrance.

### 2.2. Emulsion Polymerase Chain Reaction (ePCR)

Unlike the direct synthesis of oligonucleotide libraries to bead, ePCR enables multiple round monoclonal bead SELEX. *In vitro* compartmentalisation (IVC) using water in oil (W/O) emulsion technology was developed in 1998 [33] for the *in vitro* evolution of enzymes. In this technique, picolitre volume aqueous droplets in an oil medium are used as miniature reaction vessels. Due to their high surface area to volume ratio, each droplet could be considered the mimetic of a single cell. Single-molecule PCR reactions can be performed within each of these droplets in a process termed emulsion PCR (ePCR) [34]. Further to this, IVC can also allow for simultaneous amplification of oligonucleotide decorated monoclonal microbeads within droplets without any cross-reactivity.

ePCR is used to perform high efficiency, low error PCR when amplifying oligonucleotide libraries [35]. The first advantage of ePCR is that it limits the formation of non-specific amplicons during amplification. Non-specific amplicons are erroneous products of PCR, manifesting as nucleotide chains of exponentially increasing length. Amplicons form by assimilating free sequences and consequently reduce library diversity which, in SELEX, eliminates potential aptamer candidates. A second advantage of ePCR is that PCR efficiency is increased due to the homogenous conditions within each reaction vessel. The compartmentalisation of sequences prevents cross-contamination and as a result lowers the likelihood of detrimental chimeric strand formation and recombination. Furthermore, the homogenous nucleotide content within each droplet lessens amplification bias for shorter sequences by compartmentalising template strands of different lengths [36,37,38].

#### 2.2.1. Emulsion Formation

**Figure 2:**
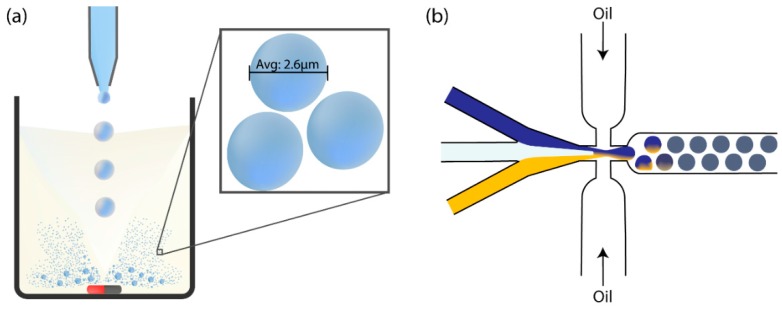
Methods of emulsion generation. (**a**) Emulsion generation using a stirrer to break the introduced aqueous phase into droplets within an oil medium; with an average reported droplet diameter of 2.6 µm [33]; (**b**) Using PDMS-based microfluidics, different reagents (Blue and Yellow) are mixed at the focal point of a mixed junction. Oil flowing in parallel interrupts the flow of reagents, which splits the stream into monodisperse droplets.

The effectiveness of ePCR, and its integration into SELEX, is dependent upon emulsion monodispersity. Emulsion generation can be performed either with stirrers or microfluidic devices (Figure 2). Both methods rely on the Poisson distribution to predict and maximise the proportion of droplets that contain exactly one sequence as a function of sequence concentration [39].

Certain methods of emulsion formation use stirrers to generate surfactant stabilised water droplets in an oil phase (Figure 2a) [34]. The primary drawback to such methods is the large variability in droplet size. High polydispersity can cause differences in activity between different droplets, and increases the uncertainty when predicting the proportion of droplets that contain exactly one sequence (the Poisson distribution model used assumes each droplet is of the same size). Minimising the number of droplets containing more than one sequence requires the use of a substantially lower sequence concentration which in turn results in a larger number of empty droplets [40].

Another approach for emulsion generation is to use microfluidic technology. Microfluidic platforms for emulsion generation typically only require a cross-junction of channels [41]. At certain junctions droplet reagents in laminar flow can be combined into droplets at regular intervals by the constant flow of an immiscible separation liquid such as silicon oil (Figure 2b). Microfluidic devices are typically made of polydimethylsiloxane (PDMS). The soft lithography involved in forming microfluidic emulsion forming devices involve the use of a clean rooms, moulds, and specialised engineers [42]. This process is more specialised than stirrer emulsion generation methods due to additional equipment and expertise required for the microfluidic droplet setup, including pumping equipment, tubing, and microscopes [41], which hinders its more widespread use.

#### 2.2.2. Uses of ePCR and Microbeads for Molecular Recognition Analysis and Aptamer Selection

The integration of beads with ePCR has a wide range of applications. These include but are not limited to: nucleotide amplification [43,44,45], aptamer selection [17,46] detection of circulating genetic material [47,48,49], detection of genetic variations [50,51], genetic analysis [52,53,54], selection of enzymatic aptamers [55] and sequencing [56,57,58,59].

**Figure 3:**
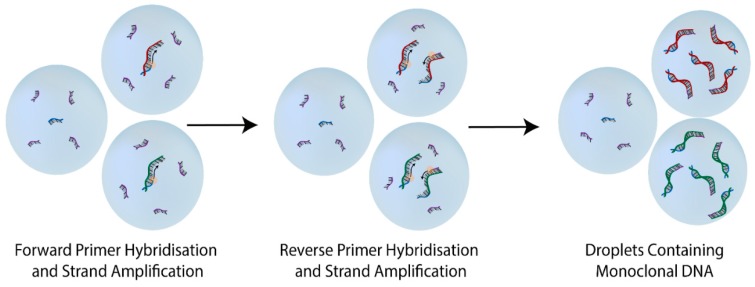
Emulsion Polymerase Chain Reaction. Droplets are formed containing PCR reagents, including forward primers (Blue strands) and reverse primers (Purple Strands) and template strands (Red and Green strands). Concentrations are adjusted to ensure that each reacting droplet contains one unique template strand according to Poisson statistics and results in a high proportion of droplets without template strands. Within droplets containing all necessary PCR reagents, DNA amplification occurs. PCR amplification is cycled until the droplets contain an optimal concentration of double stranded monoclonal DNA.

Emulsion technologies have been used for biological applications as early as 1961 [60]. The first uses of droplet compartmentalised biological reactions were in evolutionary experiments, these investigated *in vitro* linkage for the directed evolution of enzymes [33,61,62]. These experiments were fundamental to the subsequent development of ePCR (Figure 3) [34]. The first use of ePCR in aptamer selection was generation of monoclonal DNA droplets for high quality oligonucleotide libraries [36].

**Figure 4:**
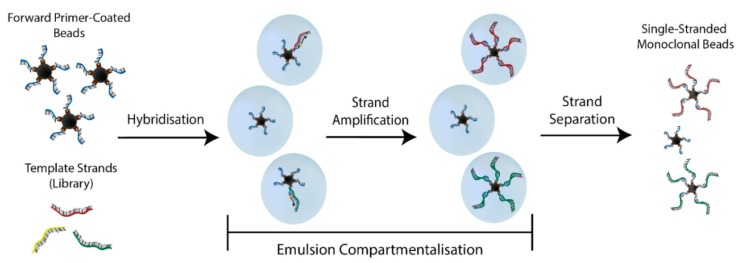
Schematic of bead-based ePCR. Step 1: Primer-coated beads and template library are combined in an emulsion, with one bead and one unique template strand per reacting droplet. Step 2: PCR is conducted within the droplets. Step 3: The emulsion is broken and double stranded library beads are magnetically collected. Step 4: By alkaline denaturation and magnetic separation single stranded monoclonal beads are formed. On average only 10% of droplets will contain sequences with as many as 80% of nucleotide coated beads being monoclonal [18].

The use of microbeads within ePCR (Figure 4) has greatly benefited the assaying of molecular binding events. In 2014 three independent groups published their utilisation of monoclonal microbeads for aptamer selection. The Yang group presented Monoclonal Surface Display SELEX [27]. Here, ePCR was used to form monoclonal beads that bind to proteins and cellular targets in the partitioning stage. These bound complexes were viewed microscopically, manually selected and then characterised. This manual selection process does not suit multiple rounds of high-throughput SELEX [27]. The Fischer group used Beads Emulsion, Amplification and Magnetics (BEAMing) for both library amplification and strand separation stages to “Just-in-time SELEX” [26]. The Soh group presented their Particle Display technique [18], which successfully integrated monoclonal beads into all stages of SELEX (Figure 1, Blue): library generation, intra-round amplification, and for FACS based partitioning and characterisation—discussed in detail in the section below.

## 3. Partitioning and Characterisation

Partitioning of binding from non-binding aptamers in classical SELEX involves target binding, washing to remove weak binders and elution of tight binders (Figure 1). All three of these partitioning steps are stochastic in nature. This can result in the loss of beneficial tight-binding aptamer sequences, especially in earlier SELEX rounds. The solution to this problem poses a significant experimental challenge of single molecule interaction detection. However by amplifying an aptamer sequence to an IVC or monoclonal oligonucleotide bead this undetectably small single molecule interaction is sufficiently amplified for the mass interaction on the bead to be detectable. In this way through IVC or monoclonal surface display, single aptamer interactions can be detected, assayed, sorted and selected in a less stochastic manner.

### 3.1. Fluorescence Activated Cell Sorting (FACS)

**Figure 5:**
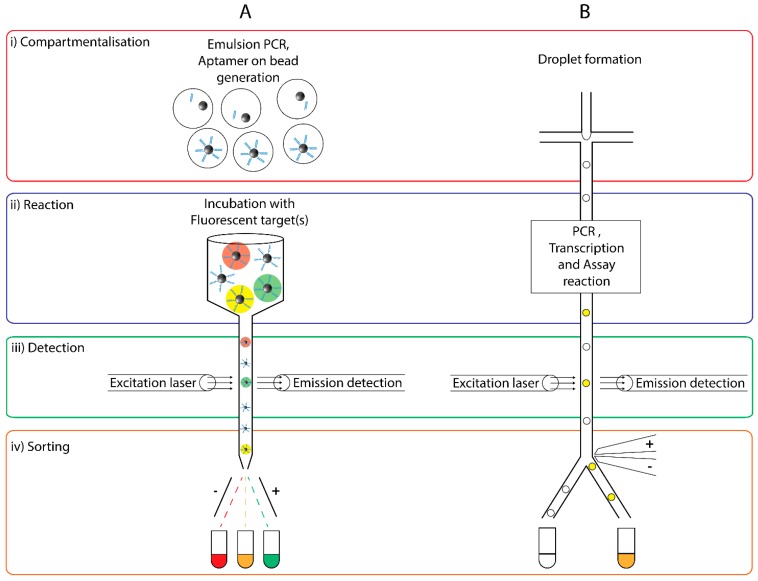
Comparison of (**A**) Fluorescence Activated Cell Sorting (FACS) and (**B**) Fluorescence Activated Droplet Sorting (FADS). (**A**) For FACS (Ai) ePCR, emulsion breaking and alkaline hydrolysis are used for generation of monoclonal aptamer bead library. (Aii) monoclonal beads are incubated with fluorescent target. (Aiii) Monoclonal beads are excited with a laser and their fluorescent emission intensities quantified. (Aiv) Beads are sorted by an electric field based upon fluorescence emission intensities (B) for FADS (Bi) Droplets are formed using microfluidics. (Bii) ePCR and transcription results in monoclonal droplets before addition of reaction reagents. (Biii) Monoclonal beads are excited with a laser and their fluorescent emission intensities quantified. (Biv) Beads are sorted by an electric field based on fluorescence emission intensities.

FACS is a technique used to rapidly sort cells or beads based on their fluorescent intensity or surface morphology (Figure 5A). Populations of cells or beads are fluorescently labelled based on a physical property and pumped into a stream through a nozzle. The nozzle breaks the stream into droplets, each containing just one cell, which is then excited with a laser beam and their emission/scattering properties measured by detectors. From these measurements, each droplet-encapsulated cell is classed based on predefined sort gates where charged electrodes alter its trajectory such that it is directed into a specified collection tube corresponding to its morphological or fluorescent properties.

FACS was invented in the late 1960s by Bonner, Sweet, Hulett and Herzenberg [24,63]. Becton Dickinson (BD) then developed commercial FACS machines which were made available in 1974 [24]. Over the next 40 years, FACS has been continuously developed and can now monitor up to 12 distinct fluorescent signals with sort speeds of up to 100,000 events per second [24]. Although originally developed for the sorting of cell populations, FACS can sort microbeads based on fluorescence. As beads are comparatively more homogenous than cells, FACS can sort beads more efficiently. As a result FACS is an ideal technology for use in selection of large libraries of bead bound aptamers against fluorescent targets. Furthermore since every member of a bead bound aptamer library is individually analysed, the stochastic nature of the binding/capture SELEX [13,64] is eliminated.

#### 3.1.1. Early Uses of FACS in Affinity Molecule Selection

The sorting power of FACS for the isolation of affinity reagents was first used to select antibodies in directed evolution experiments [65]. Yeast display is a technique where a library of antibodies is displayed on yeast cell surfaces by expression of an antibody and yeast surface fusion protein (Aga2p). Following incubation of the antibody-displaying yeast cells with fluorescent target, the cells expressing tight binding antibody could be selected using FACS [65]. Using this technique, the selection of monoclonal antibodies was possible using FACS.

#### 3.1.2. FACS Used in Aptamer Selection

With the advent of single species per bead oligonucleotide libraries [30] created by solid phase synthesis, the power of FACS could be utilised for aptamer selection for the first time. Initially FACS was just used as a post selection assay technique [32] but was later applied to the selection process [66]. As described previously, the inability to amplify these synthesised aptamer-on-bead libraries meant that only one round of selection could take place. Single round selection is a major drawback for FACS based selection techniques, although it may be possible using genetic algorithms to predict library sequences for subsequent rounds as seen in microarray aptamer selections [67].

The developments in ePCR, described in the section above, have allowed for the generation monoclonal bead libraries [34]. This process, amenable to multiple rounds of selection, harnessed the true evolutionary power of SELEX and could now be used for FACS selection. Particle display is a term coined by Wang et al in 2014 [18]. The technique involves displaying aptamers on particles or beads similarly to the other monoclonal bead techniques [27]. The fundamental difference in the particle display paper was that FACS (Figure 3A) was used to sort the monoclonal beads.

Briefly, pre-enriched libraries were amplified using ePCR to create a monoclonal bead library. This library was then incubated with biotinylated target, washed and briefly incubated with streptavidin-phycoerythrin dye before undergoing selection using FACS (Figure 5A). The top 0.1% of monoclonal beads was selected and PCR was used to recover the selected oligonucleotide aptamers from the beads. This selection process was repeated three times.

To test this new approach aptamers were isolated against the four targets thrombin, ApoE, PAI-1, and 4-1BB. Aptamers against two of these targets, ApoE and thrombin, had already been reported so a comparison could be made between classical SELEX and monoclonal bead FACS selection. Aptamers isolated using this FACS selection had 1000 times stronger binding than aptamers isolated against the same targets using traditional SELEX [18]. For thrombin, aptamers with the extraordinarily tight binding dissociation constant of 7 pM were isolated. For 4-1BB and PAI-1, previous attempts to isolate DNA aptamers against these targets had failed but here aptamers with tight binding dissociation constants of 2.32 nM and 339 pM were characterised respectively. Therefore FACS selection has a great potential to expand the range of aptamer targets currently accessible. These results show that FACS selection not only allows for the isolation of considerably tighter binding aptamers, but it can also increase the range of available targets suitable for aptamer selection.

#### 3.1.3. FACS Selection for Structure-Switching Aptamers

Monoclonal microbeads can also be used to select for structure switching aptamer beacons, initially adapted to target steroids [16]. A monoclonal bead library of aptamers was made based on a previously selected aptamer containing a three-way junction centred on a random base-binding pocket. Two complementary sequences targeting adjacent sequence positions were synthesised: one with a conjugated fluorophore and one with a conjugated quencher. Once hybridised to the aptamer library, fluorescence is turned off due to the spatial proximity of the quencher. Upon incubation of the target with the fluorophore/quencher loaded monoclonal beads, any sequences in the library that bind the target with a significant structural change will displace the quencher sequence and cause a fluorescent signal to be exhibited. The monoclonal beads can then be sorted based on this fluorescent signal using FACS thereby selecting only for structure switching aptamer beacons.

The dissociation constant of the novel structure switching aptamers was slightly stronger (4.1 µM) than that of the aptamer on which the initial library was based (5 µM) [16]. These relatively high K_d_ are understandable given the size and hydrophobicity of steroid molecules and the fact that simple unmodified DNA aptamers were used.

#### 3.1.4. Discussion of Similar Technologies

FACS has been used to select aptamers using microparticles other than simple plastic microbeads such as agarose beads and even within living cells. For selection using agarose beads, monoclonal beads containing DNA aptamer libraries had to be first developed [17]. Briefly, agarose beads containing PCR reagents and precursor DNA aptamer templates were created using a microfluidic generated water-in-oil emulsion where the water phase contains PCR reagents, DNA aptamer template and agarose. After droplet formation, a thermal cycler was used to amplify the template within the droplets and then cooled to form solid agarose beads. Oligonucleotide-containing colonies were then easily separated using SYBR Green staining. The agarose bead library could then be incubated with a fluorescently labelled target and sorted using FACS to select for the tightest binding aptamers [17]. The disadvantage of using agarose beads over ePCR oligo-coated beads is that with agarose, the oligonucleotides are not covalently linked to the bead and so dissociation can occur over time. Since FACS selection of bead libraries can take up to several hours, there is the possibility that oligonucleotides could dissociate from the agarose beads, skewing the selection results.

Cells have also been used as a medium for oligonucleotide selection using FACS. Early research into fluorescent aptamers isolated RNA aptamers against the Spinach fluorophore using classical binding/capture selection [68]. The spinach fluorophore has no fluorescence in its native state however when a ligand binds and stabilises the structure, a strong fluorescent signal is produced. By inserting this RNA aptamer sequence into a gene of interest, this spinach/RNA aptamer system can be used in microscopy for live RNA tracking, similar to how GFP protein tracking is used in molecular biology. To improve upon these results, a selection system within bacterial cells was conducted to optimise the resulting RNA aptamers for functioning in cellular environments [69]. Briefly, DNA encoding an enriched spinach fluorophore binding RNA aptamer library was cloned into plasmids. The library of plasmids were transfected into a population of *E. coli* cells, incubated with the spinach fluorophore and any cells containing fluorophore activating RNA aptamers were selected using FACS. This selection process was repeated multiple times to isolate spinach fluorophore activating RNA aptamers. This FACS selection of aptamers in the cellular environment was very successful with the new Broccoli aptamers exhibiting 2-fold higher fluorescence when expressed in *E. coli* and compared to the original Spinach aptamers [69]. Despite the success of this technique in selecting aptamers within cells, selection is limited to RNA-based aptamers.

### 3.2. Fluorescence Activated Droplet Sorting (FADS)

As previously discussed, IVC confers multiple advantages by miniaturising chemical reactions into distinct picolitre volume reaction droplets. IVC droplets have been incorporated into FACS and since each droplet encapsulates a chemical reaction, enzymatic activity can be used as a sorting criterion. Selection of this enzymatic activity through IVC and FACS has given rise to methods of fluorescently activated droplet sorting FADS.

#### 3.2.1. IVC and FADS

Within a single droplet, a range of *in vitro* reactions are possible such as transcription/translation, the expression of functional enzymes and bacterial synthesis of enzyme libraries. FADS can be used to sort the products of these reactions if fluorogenic substrates are incorporated into the reaction system. Unlike the selection techniques using FACS, which are designed to select simply for binding, enzymatic-based selection is more experimentally challenging. Fluorescent modifications, in both FACS and FADS, can lead to conformation changes of the target [70], and increase the likelihood of selecting spurious aptamers. However, many advances have been made in the field.

#### 3.2.2. From FACS to FADS: Optimising Droplet Sorting

Fluorescence-activated droplet sorting (FADS) is a method of sorting emulsion droplets similar to FACS, first introduced in 2009 [71]. The microfluidics provide an efficient way to generate monodisperse emulsion droplets, and allows for greater manipulation of both the droplet contents as well as droplet size to the femtolitre scale (Figure 2) [72]. FADS experiments have been coupled with several droplet manipulation functions, such as: droplet splitting [73,74,75], droplet fusion [74,76,77,78], and picoinjection for the addition of reagents to existing droplets [79,80,81]. Similar to the principle of FACS, FADS utilises a microfluidic system to sort emulsion droplets based on their fluorescence intensities. Therefore droplets can be sorted according to the presence or absence of enzymatic activity with the incorporation of fluorogenic substrates. Similar to FACS, sorting can be implemented by creating an electric field across the sorting junctions where emulsion droplets of defined fluorescence intensity are deflected to the designated collection tube by dielectrophoresis (Figure 5, right). Selected emulsions can then be used for downstream analysis. Sorting can be achieved at an impressive 30 kHz rate [82], although the more mature technology of FACS still holds superior sort rates [83]. For such enzymatic activity-based sorting applications, FADS eliminates the need to prepare cells and isolate them from aerosols produced in FACS sorting [84]. FADS has been used extensively in multiple applications such as the enhancement of the catalytic activity of enzymes, such as horseradish peroxidase [85], and the sorting of β-galactosidase genes in completely *in vitro* conditions [86]. This has given insight into the use of FADS to select catalytic oligonucleotides instead of simple binding aptamers.

#### 3.2.3. FADS to Select Oligonucleotides

The selection of oligonucleotides within compartmentalised droplets for improved turnover rate has been achieved for ribozymes of varying functions [87,88]. Yet the polydispersity of droplets in these methods and the confines in throughput has limited selection efficacy. FADS has been used in the selection of ribozymes [25]. In previous ribozyme evolution experiments, the procedure relied on the self-modification of the RNA library, or ribozyme attachment to a substrate, followed by the enrichment of selected RNA molecules tethered to the product [89]. The selection process is based on intramolecular reactions in single turnover conditions in which ribozyme mutants with a high turnover rate are likely to be precluded. Mutations of a ribozyme’s X-motif using error-prone PCR were used to generate a library of ribozyme mutants [25]. Individual variants were compartmentalised into monodisperse droplets along with fluorogenic substrates. In FADS, each step (e.g., library amplification, encapsulation of droplets and activity assay) is uncoupled which is suitable for directed evolution of most ribozymes working in intermolecular reactions or multiple turnover conditions [25]. The selected ribozyme variant exhibited a 28-times greater activity than the original ribozyme.

#### 3.2.4. Possible Use of FADS on Oligonucleotide Selection with Beads

Previous work demonstrated the potential of *in vitro* selection and directed evolution of ribozymes by FADS technique [25]. Given the previously discussed advantages of monoclonal beads and ePCR, the integration of catalytic nucleotide coated beads into a FADS system should allow for the selection of high-performance nucleotide enzymes. Since oligonucleotide-modified beads can be encapsulated inside microfluidic droplets [50], encapsulated oligonucleotide libraries can be easily incorporated into FADS systems [89]. Combining FADS with oligonucleotide-coated beads should be a powerful approach going forward for the aptamer field, particularly if combined with emerging XNA approaches [90].

## 4. Conclusions

Tethering oligonucleotides to beads through ePCR has increased the efficiency and versatility of established biotechnologies, including SELEX. The combination of monoclonal beads and FACS has led to robust aptamer selection techniques. One of the major disadvantages of classical binding/capture SELEX is the stochastic nature of the selection [13,64]. The use of FACS reduces this stochasticity, because every member in the library is individually assayed and sorted. This ensures that beneficial sequences are not lost and that detrimental sequences, such as nonspecific amplicons, are not preserved. Furthermore the efficiency of enrichment is substantially increased. Soh group’s FACS selection of a monoclonal bead library method resulted in a 1.7 × 10^9^ fold enrichment of an aptamer population within a single round. This is 100 million times better than the 10-fold enrichment typically observed for a classical SELEX round. This in effect lowers the number of selection rounds required and the probability of candidate loss [18]. Although other media for FACS selection have been demonstrated [17,69], aptamer beads are practical for most aptamer selection applications. Their application to exotic selection techniques involving FACS, such as for structure switching aptamer beacons [16], present significant opportunities to expand existing approaches. FADS selection offers the tantalising opportunity to select not just for binding, but for higher complexity molecular events such as enzymatic activity.
